# The Interaction between the Immune System and Epigenetics in the Etiology of Autism Spectrum Disorders

**DOI:** 10.3389/fnins.2016.00329

**Published:** 2016-07-12

**Authors:** Stefano Nardone, Evan Elliott

**Affiliations:** Faculty of Medicine, Bar Ilan UniversitySafed, Israel

**Keywords:** autism, epigenetics, immune system, maternal immune activation, DNA methylation, microbiome

## Abstract

Recent studies have firmly established that the etiology of autism includes both genetic and environmental components. However, we are only just beginning to elucidate the environmental factors that might be involved in the development of autism, as well as the molecular mechanisms through which they function. Mounting epidemiological and biological evidence suggest that prenatal factors that induce a more activated immune state in the mother are involved in the development of autism. In parallel, molecular studies have highlighted the role of epigenetics in brain development as a process susceptible to environmental influences and potentially causative of autism spectrum disorders (ASD). In this review, we will discuss converging evidence for a multidirectional interaction between immune system activation in the mother during pregnancy and epigenetic regulation in the brain of the fetus that may cooperate to produce an autistic phenotype. This interaction includes immune factor-induced changes in epigenetic signatures in the brain, dysregulation of epigenetic modifications specifically in genomic regions that encode immune functions, and aberrant epigenetic regulation of microglia. Overall, the interaction between immune system activation in the mother and the subsequent epigenetic dysregulation in the developing fetal brain may be a main consideration for the environmental factors that cause autism.

## Involvement of maternal immune activation in autism

The etiology of autism spectrum disorders (ASD) is widely defined by the interaction between genetics and environmental factors. Within the past two decades, a large number of resources have been invested in understanding the genetic underpinnings of ASD. Several large scale genetic studies have uncovered single nucleotide variations (SNVs; Gaugler et al., 2014; De Rubeis and Buxbaum, 2015; LoParo and Waldman, 2015), copy-number variations (CNVs) (Shishido et al., 2014) and *de novo* mutations (De Rubeis et al., 2014a; Iossifov et al., 2014) that are associated with ASD risk. Recent studies have found that common genetic variations account for a high percentage of the genetic risk for ASD (Gaugler et al., 2014). Of particular importance in this current discussion, the genetic variations associated with autism are also often present in individuals without an ASD diagnosis. One investigation reported that the presence of autism-risk alleles in individuals that are not diagnosed with ASD was associated with a higher IQ (Clarke et al., 2016), while another study found that genetic risk for ASD is associated with changes in social behavior even in individuals without an ASD diagnosis (Robinson et al., 2016). Overall, these studies highlight the fact that genetic risk for autism can influence multiple aspects of behavior, but it is often not sufficient to induce the full spectrum of behavior needed for a diagnosis of ASD. Therefore, a genetic contribution is likely to introduce a predisposition to develop ASD, while its actual onset requires a further environment agitation.

Current studies have been exploring the possible environmental factors that may be involved in the etiology of ASD. An expanding list includes viral infection and exposure to environmental toxins during pregnancy, gestational diabetes, among others (Grabrucker, 2012). In particular, compounding evidence supports a role for immune system activation at specific time frames in the pregnant mother as a risk factor for autism (Table 1). The first evidence for this connection was provided from the Rubella outbreak of the 1960s. Autism was diagnosed in ~5–10% of children born to mothers that were infected by Rubella virus (Chess, 1971, 1977; Hutton, 2016). Subsequently, a study, surveying Danish births from 1980 to 2005 determined a three-fold increase in the incidence of ASD in children whose mothers were hospitalized for viral infection, specifically during the first trimester of pregnancy (Atladóttir et al., 2010). A report of Swedish births found a 30% increase in ASD when the mothers were hospitalized for viral infections during pregnancy (Lee et al., 2014). However, unlike the Danish study, this investigation reported a significant effect for an association between ASD diagnosis and viral infection in all three trimesters.

**Table 1:** **Main manuscripts that investigated the association between MIA and autism, epigenetic changes in the autism postmortem brain, or MIA-induced changes in brain epigenetic patterns in mouse models**.

**Geographic region**	**Association with autism**		**References**
**MATERNAL IMMUNE ACTIVATION IN ASD-EPIDEMIOLOGICAL STUDIES**			
United States	Positive association with Congenital rubella syndrome		Chess, 1977
Denmark	Positive association with hospitalization for viral infections in first trimester and bacterial infections in second trimester		Atladóttir et al., 2010
Sweden	Positive association with hospitalization for viral infections in all three trimesters		Lee et al., 2014
Sweden	Positive association with maternal autoimmune disease in general. In addition, association with maternal Myasthenia Gravis and Rheumatic Fever		Keil et al., 2010
United States, CA	No association with maternal autoimmune diseases in general. Positive association with maternal psoriasis		Croen et al., 2005
Denmark	Positive association with maternal Rheumatoid Arthritis, Celiac Disease, and Type1 Diabetes		Atladóttir et al., 2009
Canada	Positive association with Systemic Lupus Erythematosus		Vinet et al., 2015
**Brain Area**	**Method**	**Main differences between control and autism cohorts**	**References**
**EPIGENETIC CHANGES IN ASD BRAIN-POSTMORTEM STUDIES**			
Frontal cortex, temporal cortex and cerebellum	Genome-wide 450K Illumina BeadArray	Hypomethylation in PRRT1 and C11orf21; Hypermethylation in ZFP57 and SDHAP3	Ladd-Acosta et al., 2014
Frontal and cingulate cortex	Genome-wide 450K Illumina BeadArray	Hypomethylation in genes implicated in immune response. Hypermethylation in genes implicated in synaptic processes	Nardone et al., 2014
Cerebellum and occipital cortex	Genome-wide 27K Illumina BeadArray	No significant differences	Ginsberg et al., 2012
Cerebellum and cortex	Gene-specific sodium bisulfite sequencing	Hypermethylation in Shank3	Zhu et al., 2014
Temporal cortex	Gene-specific sodium bisulfite sequencing	Hypermethylation in RELN	Lintas et al., 2016
Cerebellum	Methylation-sensitive PCR; Chip-real time PCR	Hypermethylation and increased H3K27me3 in EN-2 gene; global DNA hypermethytation	James et al., 2013
Cerebellum	hMeDIP-real time PCR; Chip-real time PCR	Hyperhydroxymethylation and decreased MeCP2 binding In EN-2 gene promoter	James et al., 2014
Frontal cortex	H3K4me3 Chip-seq on FACS-sorted neuronal nuclei	No significant differences; subset of autism cases displays spreading of H3K4me3 from transcription start sites to adjacent regions	Shulha et al., 2012
**Brain area**	**Method**	**Main findings**	**References**
**MIA-INDUCED EPIGENETIC CHANGES IN BRAIN-RODENT STUDIES**			
Hypothalamus	Gene-specific sodium bisulfite sequencing	Decrease in MECP2 and LINE1 methylation	Basil et al., 2014
Frontal cortex	Gene-specific sodium bisulfite sequencing	Increase in GAD1 and GAD2 methylation	Labouesse et al., 2015
Frontal cortex and hippocampus	Western blot; Chip-real time PCR	Global histone hypoacetylation in the cortex of juvenile offspring; promoter-specific hypoacetylation in adult cortex and hyperacetylation in adult hippocampus	Tang et al., 2013
Frontal cortex	Chip-seq	No significant changes in H3K4me3 marks	Connor et al., 2012

Additional support for the role of maternal immune activation (MIA) in the development of autism has come from animal experimentation. MIA can be simulated by injecting pregnant rodents with Poly I:C, a synthetic double-stranded mimetic of the RNA molecule, which triggers an immune response through the activation of Toll like Receptor 3, and subsequent expression of interferon-1 (Alexopoulou et al., 2001). This process induces activation of both innate and adaptive immune regulatory mechanisms in the pregnant rodent female. Offspring can then be tested for behavioral abnormalities and changes in neurodevelopment. Malvoka et al. determined that the offspring of pregnant mice treated with Poly I:C displayed all the core deficits associated with ASD including problems in social, communication, and repetitive behaviors (Malkova et al., 2012). Several follow up studies verified these findings and went on to suggest other related deficits in neurodevelopment that are responsible for this behavior (Smith et al., 2007; Canetta et al., 2016; Choi et al., 2016). In addition to rodent models, MIA has recently been induced in monkeys. Offspring of Rhesus monkeys subjected to MIA displayed deficits in social behavior and an increase in repetitive behaviors (Bauman et al., 2014; Machado et al., 2015). Therefore, both epidemiological and animal modeling studies support a causative role for MIA in the etiology of ASD.

Other studies have shown another possible trigger of an immunity-induced autism phenotype could be due to the presence of an autoimmune disorder in the mother. A study of the Swedish health registry uncovered an increase of 60% in the odds for developing autism among those children of mothers diagnosed with an autoimmune disorder (Keil et al., 2010). Interestingly, an increase of 40% was detected among children whose fathers had an autoimmune disorder. A separate study in Northern California found an association between maternal psoriasis and the insurgence of autism, although the association between autoimmune diseases in general and autism was not significant (Croen et al., 2005). In a large study examining nearly 700,000 Swedish births, Atladottir et al. found that autism was associated with maternal Rheumatoid Arthritis and Celiac Disease, as well as those with a family history of Type 1 Diabetes (Atladóttir et al., 2009). An increase in autism was also identified in a cohort of Canadian children from mothers with Systemic Lupus Erythematosus (Vinet et al., 2015). A recent meta-analysis of studies performed in multiple locations confirmed a significant association between maternal autoimmune diseases and ASD (Chen et al., 2016). Generally, it has become apparent that both MIA and maternal autoimmune disease are associated with ASD.

## Epigenetics in autism

Epigenetics is responsible for the proper development of the nervous system and is highly regulated by environmental factors, such as an inflammatory response (Jirtle and Skinner, 2007). Epigenetics refers to heritable changes in gene regulatory mechanisms that are independent from alteration of the underlying DNA sequence. Two of the most commonly studied epigenetic markers include DNA methylation and histone modifications (Jones, 2001; Goldberg et al., 2007). Both markers influence the establishment of gene transcription patterns through multiple mechanisms, including the regulation of genomic, structure, and the accessibility of genomic loci to diverse regulatory factors (e.g., transcription factors, enhancers, silencers). Temporal changes in epigenetic signature during the developmental stage finely tunes the differentiation of precursor cells into their specific mature state (Kiefer, 2007). Therefore, epigenetic markers display a relatively high level of plasticity during periods of cellular specification, including in brain development (Spiers et al., 2015). As such, it is likely that environmental disturbances during pregnancy may induce stable and long-term modifications in epigenetic patterns that probably survive until adulthood.

Genetic studies demonstrated preliminary evidence of a role for epigenetic mechanisms in the etiology of ASD. Analysis of hundreds of genes associated with ASD reveal enrichment for two biological functions: synaptic plasticity or chromatin binding factors (Lasalle, 2013; De Rubeis et al., 2014b). The enrichment of chromatin binding genes in autism studies suggests their potential role in the etiology of ASD. Rett Syndrome is one very well-known example of a genetic neurodevelopmental condition that includes autistic behavior, and whose etiology is directly related to epigenetic regulation. Rett Syndrome is caused by mutations in the gene MECP2, which encodes for the methylated DNA binding protein MeCP2 (Amir et al., 1999). Upon binding methylated DNA, MeCP2 either activates or inhibits gene transcription depending on the genomic context (Chahrour et al., 2008). The fact that mutations in the MECP2 gene induce a syndrome with autistic-like behavior, indicates a direct link between DNA methylation-regulated gene expression and ASD symptomatology. Other chromosome binding genes that have been found to be strongly associated with ASD include CHD8, MBD5, and AUTS2 (Sultana et al., 2002; Cukier et al., 2012; Bernier et al., 2014). Therefore, genetic studies have long proposed an involvement of epigenetics in the etiology of ASD.

While genetic studies have provided preliminary evidence for the involvement of epigenetics in the etiology of ASD, epigenetic-based studies were necessary to finally prove a direct association between dysregulation of epigenetic signatures, both in peripheral tissues and the brain, and ASD (Ciernia and LaSalle, 2016). Post-mortem brain epigenetic studies have been critical in understanding how epigenetic patterns may be affected in the ASD brain. This is due to the fact that each tissue and cell type are characterized by highly specific epigenetic signatures. Accordingly, the interrogation of peripheral tissues may not truly represent changes seen in brain tissues. Previous reviews have thoroughly discussed the literature on epigenetic dysregulation in ASD, including the periphery (Tordjman et al., 2014; Loke et al., 2015; Ciernia and LaSalle, 2016). Here, we will focus specifically on epigenetic dysregulation in the brain, in order to later discuss the possible role of MIA in brain epigenetics and development of ASD (Table 1).

Several gene-specific studies have been performed to provide preliminary evidence of DNA methylation dysregulation in the ASD brain. An initial study analyzed the DNA methylation profile of the autism-related gene Shank3. This study determined a hypermethylation of multiple intragenic regions in the Shank3 gene (Zhu et al., 2014). While mutations in Shank3 may be found in ~0.5% of individuals with autism, this study suggests that epigenetic changes in Shank3 may actually be more widespread. Therefore, epigenetic alterations may significantly affect the function of autism-related genes in the absence of those rare genetic changes. Other studies have also determined dysregulated DNA methylation signatures in the autism-related genes RELN and EN-2 (James et al., 2013, 2014; Lintas et al., 2016).

To date, only a few epigenetic studies have investigated in a genome-wide fashion the DNA methylation profile of post-mortem brain tissue from individuals with ASD diagnosis and matched controls. These studies have been performed using the Illumina 450K BeadArray, which profiles the methylation level of ~480,000 CpGs throughout the entire human genome. Using this method, Ladd Acosta et al. discovered four differentially methylated regions in the DNA when comparing the control and autism groups (Ladd-Acosta et al., 2014). These methylated regions containing multiple CpGs included the genes PRRT1, ZFP57, and TSPAN32 in the cortex, and SDHAP3 in the cerebellum. In a separate study, we found that over 5000 individual CpGs were differentially methylated in the frontal cortex when comparing the control and autism cohorts (Nardone et al., 2014). Hypermethylated and hypomethylated CpGs were mainly found in genomic regions that were associated to genes enriched with synaptic functions and immune response, respectively. Of interest, the gene TSPAN32 had about an 8% decrease in DNA methylation levels in the ASD vs. control group in both studies. An additional genome-wide study investigating the DNA methylation in both the cerebellum and occipital cortex found no significant changes between the control and autism groups, suggesting that the effects in the brain are region-specific (Ginsberg et al., 2012). Overall, these findings corroborate the aforementioned gene-specific studies, further reinforcing the evidence for an involvement of epigenetics in the etiology of ASD. In addition, they highlight the brain-area specificity of epigenetic signature, pinpointing the forebrain as one of the brain regions more liable to environmental insults.

## Autism and epigenetic regulation of immune genes and immune cells

There is converging evidence that the epigenetic regulation of the immune system may be involved in the etiology of ASD. First, as mentioned above, hypomethylated CpGs detected in frontal cortex of autistic individuals were enriched in genes involved in immune response (Nardone et al., 2014). The genes associated to those CpGs displayed a significant increase in their transcription: of particular interest were C1qA, C3, TNF-alpha, and other transcription factors that are known to be essential in the development of the microglia. These findings correlate very well with genome-wide transcriptomic studies that demonstrated an increased expression of microglia and innate immune response-related genes in the brain of individuals with autism (Voineagu et al., 2011; Gupta et al., 2014). Therefore, there is evidence for dysregulation of the immune response in the autism brain at both the level of gene transcription and DNA methylation.

Mouse studies have demonstrated that the malfunctioning of the epigenetic machinery, specifically in microglia cells, can cause autistic-like behavior. Much of this evidence stems from studies on Rett Syndrome and the MECP2 gene. As aforementioned, MeCP2 binds methylated CG sites and contributes to gene transcription regulation. A major study demonstrated that a Rett Syndrome-like phenotype, present in microglia-specific MECP2 KO mice, could be reversed by replenishing the MECP2 KO with wild type microglia (Derecki et al., 2012). Furthermore, Maezawa et al. proved that deletion of MECP2 in microglia induced dysregulation of extracellular glutamate levels and neuronal dendrites (Maezawa and Jin, 2010). In a follow-up study, the same research group demonstrated that inhibition of the interaction between microglia and neurons in the MECP2 KO mice can attenuate many of the Rett Syndrome-like behavioral manifestations (Horiuchi et al., 2016). These studies suggest that MeCP2 influences mouse behavior by regulating epigenetic machinery in microglia. It is important to note, however, that a recent study was not able to entirely replicate those main findings (Wang et al., 2015). So it remains unclear whether microglia is the main or only one of many factors that play a role in the etiology of Rett Syndrome. while the exact role of microglia is not completely defined, there is much evidence to suggest the epigenetic regulation of microglia plays an important role in the etiology of ASD.

Although there is sufficient evidence for increased immune activation in the brains of autistic subjects, immune system genes are not among those that are often mutated in autism. Therefore, immune activation is unlikely to be explained by genetic etiology, but rather via epigenetic machinery. In conclusion, the immune activation detected in brain and blood samples of autistic subjects may be due to environmental factors and mediated by epigenetic mechanisms.

## Can the prenatal environment induce DNA methylation changes in the offspring?

The primary question that we aim to address is whether the epigenetic differences detected in the brain and peripheral tissues of autism individuals are due to environmental insults during development, such as MIA. In order to consider this possibility, we must first establish the extent to which the prenatal environment may generally affect the epigenetic machinery. Multiple human studies have been performed to determine a link between environmental insults during pregnancy and altered methylation patterns in the offspring. A well-known example is the study based on the Dutch Famine Winter, which investigated the offspring of mothers exposed to famine conditions during pregnancy. The offspring displayed dysregulation of DNA methylation in genomic regions associated to the gene IGF2, an imprinted gene that has primary roles in metabolism (Heijmans et al., 2008). This epigenetic alteration was maintained for several decades after the famine, showing that environmental insults during pregnancy may have a very longlasting effects on epigenetic patterning. Similarly, multiple studies have found dysregulation in DNA methylation in the cord blood of offspring from mothers diagnosed with gestational diabetes (Quilter et al., 2014; Finer et al., 2015). In addition, other environmental cues that can influence the stress response during pregnancy and the insurgency of maternal depression episodes (Nemoda et al., 2015) have been associated with DNA methylation changes in offspring. Yet some of the most meaningful data came from studies investigating the effects of maternal smoking. Maternal smoking during pregnancy has been associated with dysregulation of DNA methylation in multiple genes in newborns, including NeuroG1 and CNTNAP2, which are known to play a pivotal role in language development and have been associated with ASD by several studies (Küpers et al., 2015; Lee et al., 2015; Richmond et al., 2015). An additional factor that has the potential to influence the epigenetic machinery is the level of maternal serum folate during pregnancy. Folic acid deficiency during pregnancy may be accountable for the occurrence of neurodevelopmental abnormalities, such as spina bifida. Nowadays folic acid is a widely-prescribed supplement during pregnancy, and has been associated with a decreased risk of developing ASD, despite its mechanism of action not being completely understood (Schmidt et al., 2012; Surén et al., 2013). Recently, it has been shown that a mother's folate level is correlated to differential methylation in several developmental-related genes in offspring (Joubert et al., 2016). As such folic acid supplementation may be an additional environmental factor that regulates epigenetic programming during development. Together, these human studies provide a firm basis for the linkage between the *in utero* environment and dysregulation of DNA methylation patterning.

## Could MIA be responsible for epigenetic dysregulation in ASD?

Considering the association of both MIA and epigenetic dysregulation in autism, immune activation in pregnant mothers may be instrumental in epigenetic dysregulation and the downstream behavioral phenotypes observed in autistic children. In support of this idea, recent animal studies have proven that MIA can alter epigenetic patterns associated to autism-candidate genes in the offspring brain (Table 1). An initial study found that MIA induced a decrease in global histone acetylation levels in the cortex of juvenile offspring. Also detected were gene-specific changes of histone acetylation at several neuron-related gene promoters in both the cortex and hippocampus of juvenile offspring (Tang et al., 2013). Although these epigenetic patterns did not survive till adulthood, it is conceivable that their transient effect on gene transcription may cause permanent effects that persist into adulthood. The authors did in fact show that adult offspring displayed abnormalities in exploratory behavior. A separate study found only few changes in histone acetylation levels in adult mice after MIA (Connor et al., 2012). So it appears most likely that changes in histone modifications occur mainly during developmental stages and are no longer present in adulthood. Preliminary studies have recently been performed in order to investigate the relationship between MIA and DNA methylation in the brain of offspring. The injection of pregnant mice with Poly I:C induced a decrease of DNA methylation of CpGs associated to MECP2 and LINE-1 in the hypothalamus of the newborns (Basil et al., 2014). While the biological meaning of dysregulation in MECP2 is well understood, the implications of LINE-1 dysregulation remainunclear, especially in light of the fact that LINE-1 methylation levels are often employed to detect any variation in global DNA methylation. Further studies are necessary to fully understand if a decrease in DNA methylation detected in LINE-1 can be indicative of genome-wide hypomethylation after MIA. In a more recent study, offspring of Poly I:C treated mothers displayed hypermethylation at the promoter of Glutamic Acid Decarboxylase 1 (GAD1) and Glutamic Acid Decarboxylase 2 (GAD2) genes in the prefrontal cortex (Labouesse et al., 2015). An increase in DNA methylation levels was associated with augmented MeCP2 binding and lower gene expression. GAD1 and GAD2 are responsible for the production of the inhibitory neurotransmitter Gamma-Amino Butyric acid (GABA) from its precursor Glutamate. Downregulation of the GABAergic system has been characterized extensively in both autistic children and animal models. Levels of GABA and its receptors have been found to be downregulated in the cortex of individuals with autism (Harada et al., 2011; Crider et al., 2014), and pharmacological activation of GABA receptors improved social behavior in the BTBR autism mouse model (Han et al., 2014). Therefore, the link between MIA and regulation of GABAergic gene-expression operated by DNA methylation, can represent a possible mechanism leading toward GABAergic dysfunction in ASD. In summary, animal studies have provided evidence that MIA induces long term changes in DNA methylation patterns in the brain of offspring.

Although there is an extreme complexity in conducting such studies, there is a need for a human investigation of the relationship between MIA and epigenetics in young children. However, other environmental factors that have been associated with autism—and are known to affect the epigenetic machinery—can also regulate the maternal immune response during pregnancy. For example, as mentioned earlier, gestational diabetes has been associated with ASD, and has been correlated with increased immune activation in the mother and dysregulated placental DNA methylation. Therefore, it is plausible that environmental cues such as gestational diabetes can affect epigenetics modulating the activation of immune system.

## Mechanisms through which the immune response may affect epigenetic patterning

If immune system activation leads to epigenetic dysregulation seen in autism, there should be a direct molecular mechanism that links the two phenomena. Animal studies have focused on determining the specific immune factors that might be responsible for the MIA-induced autism phenotype. Smith et al. showed the inhibition of the maternal IL-6 mediated pathway attenuates the MIA-induced autism behavior in offspring (Smith et al., 2007). Maternal IL-6 was directly responsible for MIA-induced gene transcription changes in the offspring frontal cortex. In addition, it has been previously demonstrated that IL-6 is transferred across the human placenta (Zaretsky et al., 2004), which suggests that maternal IL-6 could directly affect the development of the fetal brain. Multiple studies have independently determined that IL-6 can activate DNA methyltransferase 1 (DNMT1), thus providing a direct interaction between MIA and epigenetic regulation. IL-6 induces the nuclear translocation of DNMT1 through AKT-dependent phosphorylation of a nuclear location sequence on the DNMT1 protein (Hodge et al., 2007). IL-6 triggers the DNMT1-mediated hypermethylation at specific promoters in cancer cell lines, that in turn increases the cellular growth rate along with other oncogenic properties. While these effects were witnessed in cancer cells, it is likely that IL-6 can regulate DNMT1 in other cell types such as brain cells, particularly during development. DNMT1 is considered a maintenance methyltransferase, which functions mostly to maintain methylation patterns during cellular proliferation. As such, it is most likely that IL-6-induced DNMT1 activation would have effects during neurogenesis, which takes place mostly during developmental time periods, such as *in utero*. Therefore, there may be a direct interaction between IL-6 and epigenetic machinery, which in turn can regulate gene expression after MIA. A recent study has found that IL-17 activation is also mandatory for an MIA-induced autism-like phenotype in mice (Choi et al., 2016). In fact, it was demonstrated that IL-17-producing T-cells were recruited to the placenta and IL-17 was activated in the developing neocortex. Possible effects of IL-17 on epigenetic machinery have not been extensively studied, however, one study did find that IL-17 inhibits HDAC activity, most likely through PI-3Kinase signaling pathway (Zijlstra et al., 2012). So while IL-6 may directly mediate changes in DNA methylation, IL-17 may affect histone acetylation. Further investigations are still required to really understand if the effect of prenatal IL-6 and IL-17 on offspring behavior is mediated through epigenetic mechanisms.

The mechanism by which most cytokines are likely to influence epigenetic patterns is through regulation of signal transduction pathways that activate epigenetic enzymes or recruit chromatin regulators to the DNA. Two of the main signal transduction pathways that are activated by cytokines are JAK/STAT and MAPK/ERK signaling pathways (Heinrich et al., 2003). STAT proteins act as transcription factors, and also mediate the remodeling of histone acetylation at STAT-binding sites (Wei et al., 2010). The MAP/ERK signaling pathway affects multiple epigenetic modulators, such as CREB. The activation of CREB by phosphorylation involves the recruitment of a chromatin modifying complex, the histone acetyltransferase CBP to the chromatin (Ogryzko et al., 1996). CREB has been shown to be activated by various interleukins, including IL-18 (Zhou et al., 2014) and IL-6 (Melemedjian et al., 2014). While we have just shown that two specific pathways through which IL-6 and IL-17 may affect epigenetic enzymes, it is probable that cytokines or other soluble mediators of the immune response can actually affect epigenetic marks through multiple signaling pathways.

## The microbiome as a possible modulator between MIA and the autism phenotype

Of notable interest to neuroscience in general, and to neurodevelopment in particular, is the contribution of the microbiome to human health. The population of bacteria that lives in symbiosis with the human body, collectively known as the microbiome, has recently been shown to influence behavior (Cryan and Dinan, 2012). Of particular interest to this review, the microbiome conducts a very tight crosstalk with the host immune system (Kau et al., 2011). In fact, the microbiome population affects the activity of the immune system, and vice-versa. In addition, recent studies have found that autistic individuals own a distinct microbiome signature compared to non-autistic individuals and even to individuals who were previously diagnosed with Pervasive Developmental Disorder (De Angelis et al., 2013). A recent rodent study suggested that MIA-induced autism phenotypes are caused by an alteration in the number and composition of the wild type mouse microbiota; in particular *B. fragilis* played a fundamental role (Hsiao et al., 2013). Hsiao et al. found that *B. fragilis* specifically affects repetitive behaviors while not having any influence on social behaviors. Therefore, MIA may affect the development of ASD by partially regulating the microbiome composition, even though the molecular mechanisms by which the microbiome may trigger ASD-related behavior are still unknown.

One of the main metabolic products of the microbiome is short-chained fatty acids, including sodium butyrate. Sodium butyrate is well characterized as a histone deacetylase (HDAC) inhibitor (Davie, 2003), and can easily cross the blood-brain barrier. Therefore, sodium butyrate could represent a direct link between the microbiome and epigenetic machinery in the brain. In a previous study it was found that sodium butyrate can also attenuate autism-like behavior in the BTBR mice, which is another autism mouse model often used in pharmacological studies (Kratsman et al., 2015). It was also shown that sodium butyrate specifically affected the transcription of genes involved in the excitation/inhibition balance and GABAergic signaling in mouse prefrontal cortex. So aside from being a common bacterial metabolite, sodium butyrate, is also a very efficient epigenetic modulator that can possibly influence processes that rely on a subtle balance of neurotransmitters. Further studies are needed to understand if immune regulation of the microbiome can induce epigenetic changes in the brain through modulation of fatty acids.

## Conclusions

Separate studies have implicated both immune and epigenetic dysregulation in the etiology of ASD. We set out to review the evidence indicating that the two phenomena are not independent, and that there could actually be a causal relationship between them. This relationship may have different aspects including immune activation-induced alterations in epigenetic patterns in the fetus brain, and epigenetic regulation of immune cells and immune-related genes in the brain. As was pointed out early in this discussion, the development of ASD is a multifactorial process and it is therefore possible that a genetic predisposition increases the likelihood of developing ASD after MIA. In support of this statement, one study found that MIA induced a phenotype characterized by strong social impairment specifically in mice overexpressing a double negative form of DISC1 (Ibi et al., 2010), a gene strongly associated to Schizophrenia and ASD (Ishizuka et al., 2006; Crepel et al., 2010; Zheng et al., 2011; Turner et al., 2016). In addition, a recent study found that autistic individuals who bear both CNVs and a history of MIA displayed more severe social deficits and repetitive behaviors (Mazina et al., 2015). This means MIA-induced epigenetic changes and genetic susceptibility are likely required to interact to promote autistic behavior. The study, by means of a simplistic example of GxE interaction, puts forth a two-hit model that combines the genetic makeup, as predisposing factor, and environmental cues, as trigger, for explaining the etiology of complex diseases, such in psychiatry.

There are many follow-up studies that are needed to further establish and understand the model of MIA-induced epigenetic changes in the etiology of ASD. In particular, studies are needed that look at the epigenetic studies of children whose mother's experienced MIA during pregnancy. In addition, it is necessary to understand why GABAergic genes seem to be particularly susceptible to MIA and to epigenetic modulation in the brain. Looking to the future, these studies may lead to a more integrated model of ASD biology and etiology, which will help determine the exact relationships between immune response activation and epigenetic regulation, and how they lead to specific autism phenotypes.

## Author contributions

SN and EE jointly wrote the manuscript and edited the manuscript.

## Funding

We hold grants from the Israel Science Foundation (1047/12) and Teva Pharmaceutical Industries.

### Conflict of interest statement

The authors declare that the research was conducted in the absence of any commercial or financial relationships that could be construed as a potential conflict of interest.

## References

[B1] Alexopoulou L. Holt A. C. Medzhitov R. Flavell R. A. (2001). Recognition of double-stranded RNA and activation of NF-kappaB by Toll-like receptor 3. Nature 413, 732–738. 10.1038/35099560 11607032

[B2] Amir R. E. Van den Veyver I. B. Wan M. Tran C. Q. Francke U. Zoghbi H. Y. (1999). Rett syndrome is caused by mutations in X-linked MECP2, encoding methyl-CpG-binding protein 2. Nat. Genet. 23, 185–188. 10.1038/13810 10508514

[B3] Atladóttir H. O. Pedersen M. G. Thorsen P. Mortensen P. B. Deleuran B. Eaton W. W.. (2009). Association of family history of autoimmune diseases and autism spectrum disorders. Pediatrics 124, 687–694. 10.1542/peds.2008-2445 19581261

[B4] Atladóttir H. O. Thorsen P. Øtergaard L. Schendel D. E. Lemcke S. Abdallah M.. (2010). Maternal infection requiring hospitalization during pregnancy and autism spectrum disorders. J. Autism Dev. Disord. 40, 1423–1430. 10.1007/s10803-010-1006-y 20414802

[B5] Basil P. Li Q. Dempster E. L. Mill J. Sham P. C. Wong C. C. Y.. (2014). Prenatal maternal immune activation causes epigenetic differences in adolescent mouse brain. Transl. Psychiatry 4, e434. 10.1038/tp.2014.80 25180573 PMC4203009

[B6] Bauman M. D. Iosif A. M. Smith S. E. P. Bregere C. Amaral D. G. Patterson P. H. (2014). Activation of the maternal immune system during pregnancy alters behavioral development of rhesus monkey offspring. Biol. Psychiatry 75, 332–341. 10.1016/j.biopsych.2013.06.025 24011823 PMC6782053

[B7] Bernier R. Golzio C. Xiong B. Stessman H. A. Coe B. P. Penn O.. (2014). Disruptive CHD8 mutations define a subtype of autism early in development. Cell 158, 263–276. 10.1016/j.cell.2014.06.017 24998929 PMC4136921

[B8] Canetta S. Bolkan S. Padilla-Coreano N. Song L. J. Sahn R. Harrison N. L.. (2016). Maternal immune activation leads to selective functional deficits in offspring parvalbumin interneurons. Mol. Psychiatry 10.1038/mp.2015.222 26830140 PMC4914410

[B9] Chahrour M. Jung S. Y. Shaw C. Zhou X. Wong S. T. C. Qin J.. (2008). MeCP2, a key contributor to neurological disease, activates and represses transcription. Science 320, 1224–1229. 10.1126/science.1153252 18511691 PMC2443785

[B10] Chen S. W. Zhong X. S. Jiang L. N. Zheng X. Y. Xiong Y. Q. Ma S. J.. (2016). Maternal autoimmune diseases and the risk of autism spectrum disorders in offspring: a systematic review and meta-analysis. Behav. Brain Res. 296, 61–69. 10.1016/j.bbr.2015.08.035 26327239

[B12] Chess S. (1971). Autism in children with congenital rubella. J. Autism Child. Schizophr. 1, 33–47. 5172438 10.1007/BF01537741

[B11] Chess S. (1977). Follow-up report on autism in congenital rubella. J. Autism Child. Schizophr. 7, 69–81. 576606 10.1007/BF01531116

[B13] Choi G. B. Yim Y. S. Wong H. Kim S. Kim H. Kim S. V.. (2016). The maternal interleukin-17a pathway in mice promotes autismlike phenotypes in offspring. Science 351, 933–939. 10.1126/science.aad0314 26822608 PMC4782964

[B14] Ciernia A. V. LaSalle J. (2016). The landscape of DNA methylation amid a perfect storm of autism aetiologies. Nat. Rev. Neurosci. 17, 411–423. 10.1038/nrn.2016.41 27150399 PMC4966286

[B15] Clarke T. K. Lupton M. K. Fernandez-Pujals A. M. Starr J. Davies G. Cox S.. (2016). Common polygenic risk for autism spectrum disorder (ASD) is associated with cognitive ability in the general population. Mol. Psychiatry 21, 419–425. 10.1038/mp.2015.12 25754080 PMC4759203

[B16] Connor C. M. Dincer A. Straubhaar J. Galler J. R. Houston I. B. Akbarian S. (2012). Maternal immune activation alters behavior in adult offspring, with subtle changes in the cortical transcriptome and epigenome. Schizophr. Res. 140, 175–184. 10.1016/j.schres.2012.06.037 22804924 PMC3568668

[B17] Crepel A. Breckpot J. Fryns J. P. De la Marche W. Steyaert J. Devriendt K.. (2010). DISC1 duplication in two brothers with autism and mild mental retardation. Clin. Genet. 77, 389–394. 10.1111/j.1399-0004.2009.01318.x 20002455

[B18] Crider A. Pandya C. D. Peter D. Ahmed A. O. Pillai A. (2014). Ubiquitin-proteasome dependent degradation of GABAAα1 in autism spectrum disorder. Mol. Autism 5, 45. 10.1186/2040-2392-5-45 25392730 PMC4228821

[B19] Croen L. A. Grether J. K. Yoshida C. K. Odouli R. Van de Water J. (2005). Maternal autoimmune diseases, asthma and allergies, and childhood autism spectrum disorders: a case-control study. Arch. Pediatr. Adolesc. Med. 159, 151–157. 10.1001/archpedi.159.2.151 15699309

[B20] Cryan J. F. Dinan T. G. (2012). Mind-altering microorganisms: the impact of the gut microbiota on brain and behaviour. Nat. Rev. Neurosci. 13, 701–712. 10.1038/nrn3346 22968153

[B21] Cukier H. N. Lee J. M. Ma D. Young J. I. Mayo V. Butler B. L.. (2012). The expanding role of MBD genes in autism: identification of a MECP2 duplication and novel alterations in MBD5, MBD6, and SETDB1. Autism Res. 5, 385–397. 10.1002/aur.1251 23055267 PMC3528798

[B22] Davie J. R. (2003). Inhibition of histone deacetylase activity by butyrate. J. Nutr. 133, 2485S–2493S. 12840228 10.1093/jn/133.7.2485S

[B23] De Angelis M. Piccolo M. Vannini L. Siragusa S. De Giacomo A. Serrazzanetti D. I. (2013). Fecal microbiota and metabolome of children with autism and pervasive developmental disorder not otherwise specified. PLoS ONE 8:e76993 10.1371/journal.pone.0076993 24130822 PMC3793965

[B24] Derecki N. C. Cronk J. C. Lu Z. Xu E. Abbott S. B. G. Guyenet P. G.. (2012). Wild-type microglia arrest pathology in a mouse model of Rett syndrome. Nature 484, 105–109. 10.1038/nature10907 22425995 PMC3321067

[B25] De Rubeis S. Buxbaum J. D. (2015). Genetics and genomics of autism spectrum disorder: embracing complexity. Hum. Mol. Genet. 24, R24–R31. 10.1093/hmg/ddv273 26188008 PMC4675826

[B26] De Rubeis S. He X. Goldberg A. P. Poultney C. S. Samocha K. Ercument Cicek A.. (2014a). Synaptic, transcriptional and chromatin genes disrupted in autism. Nature 515, 209–215. 10.1038/nature13772 25363760 PMC4402723

[B27] De Rubeis S. He X. Goldberg A. P. Poultney C. S. Samocha K. Ercument Cicek A.. (2014b). Synaptic, transcriptional and chromatin genes disrupted in autism. Nature 515, 209–215. 10.1038/nature13772 25363760 PMC4402723

[B28] Finer S. Mathews C. Lowe R. Smart M. Hillman S. Foo L.. (2015). Maternal gestational diabetes is associated with genome-wide DNA methylation variation in placenta and cord blood of exposed offspring. Hum. Mol. Genet. 24, 3021–3029. 10.1093/hmg/ddv013 25634562

[B29] Gaugler T. Klei L. Sanders S. J. Bodea C. A. Goldberg A. P. Lee A. B.. (2014). Most genetic risk for autism resides with common variation. Nat. Genet. 46, 881–885. 10.1038/ng.3039 25038753 PMC4137411

[B30] Ginsberg M. R. Rubin R. A. Falcone T. Ting A. H. Natowicz M. R. (2012). Brain transcriptional and epigenetic associations with autism. PLoS ONE 7:e44736. 10.1371/journal.pone.0044736 22984548 PMC3440365

[B31] Goldberg A. D. Allis C. D. Bernstein E. (2007). Epigenetics: a landscape takes shape. Cell 128, 635–638. 10.1016/j.cell.2007.02.006 17320500

[B32] Grabrucker A. M. (2012). Environmental factors in autism. Front. Psychiatry 3:118. 10.3389/fpsyt.2012.00118 23346059 PMC3548163

[B33] Gupta S. Ellis S. E. Ashar F. N. Moes A. Bader J. S. Zhan J.. (2014). Transcriptome analysis reveals dysregulation of innate immune response genes and neuronal activity-dependent genes in autism. Nat. Commun. 5, 5748. 10.1038/ncomms6748 25494366 PMC4270294

[B34] Han S. Tai C. Jones C. J. Scheuer T. Catterall W. A. (2014). Enhancement of inhibitory neurotransmission by GABAA receptors having ??2,3-subunits ameliorates behavioral deficits in a mouse model of autism. Neuron 81, 1282–1289. 10.1016/j.neuron.2014.01.016 24656250 PMC4079471

[B35] Harada M. Taki M. M. Nose A. Kubo H. Mori K. Nishitani H.. (2011). Non-invasive evaluation of the GABAergic/glutamatergic system in autistic patients observed by MEGA-editing proton MR spectroscopy using a clinical 3 tesla instrument. J. Autism Dev. Disord. 41, 447–454. 10.1007/s10803-010-1065-0 20652388

[B36] Heijmans B. T. Tobi E. W. Stein A. D. Putter H. Blauw G. J. Susser E. S.. (2008). Persistent epigenetic differences associated with prenatal exposure to famine in humans. Proc. Natl. Acad. Sci. U.S.A. 105, 17046–17049. 10.1073/pnas.0806560105 18955703 PMC2579375

[B37] Heinrich P. C. Behrmann I. Haan S. Hermanns H. M. Müller-Newen G. Schaper F. (2003). Principles of interleukin (IL)-6-type cytokine signalling and its regulation. Biochem. J. 374, 1–20. 10.1042/BJ20030407 12773095 PMC1223585

[B38] Hodge D. R. Cho E. Copeland T. D. Guszczynski T. Yang E. Seth A. K.. (2007). IL-6 enhances the nuclear translocation of DNA cytosine-5-methyltransferase 1 (DNMT1) via phosphorylation of the nuclear localization sequence by the AKT kinase. Cancer Genomics Proteomics 4, 387–398. 18204201

[B39] Horiuchi M. Smith L. Maezawa I. Jin L. W. (2016). CX3CR1 ablation ameliorates motor and respiratory dysfunctions and improves survival of a Rett syndrome mouse model. Brain. Behav. Immun. 10.1016/j.bbi.2016.02.014 26883520 PMC5531048

[B40] Hsiao E. Y. McBride S. W. Hsien S. Sharon G. Hyde E. R. McCue T.. (2013). Microbiota modulate behavioral and physiological abnormalities associated with neurodevelopmental disorders. Cell 155, 1451–1463. 10.1016/j.cell.2013.11.024 24315484 PMC3897394

[B41] Hutton J. (2016). Does rubella cause autism: a 2015 reappraisal? Front. Hum. Neurosci. 10:25. 10.3389/fnhum.2016.00025 26869906 PMC4734211

[B42] Ibi D. Nagai T. Koike H. Kitahara Y. Mizoguchi H. Niwa M.. (2010). Combined effect of neonatal immune activation and mutant DISC1 on phenotypic changes in adulthood. Behav. Brain Res. 206, 32–37. 10.1016/j.bbr.2009.08.027 19716847 PMC2846597

[B43] Iossifov I. O'Roak B. J. Sanders S. J. Ronemus M. Krumm N. Levy D.. (2014). The contribution of *de novo* coding mutations to autism spectrum disorder. Nature 515, 216–221. 10.1038/nature13908 25363768 PMC4313871

[B44] Ishizuka K. Paek M. Kamiya A. Sawa A. (2006). A review of Disrupted-In-Schizophrenia-1 (DISC1): neurodevelopment, cognition, and mental conditions. Biol. Psychiatry 59, 1189–1197. 10.1016/j.biopsych.2006.03.065 16797264

[B45] James S. J. Shpyleva S. Melnyk S. Pavliv O. Pogribny I. P. (2013). Complex epigenetic regulation of engrailed-2 (EN-2) homeobox gene in the autism cerebellum. Transl. Psychiatry 3, e232. 10.1038/tp.2013.8 23423141 PMC3590998

[B46] James S. J. Shpyleva S. Melnyk S. Pavliv O. Pogribny I. P. (2014). Elevated 5-hydroxymethylcytosine in the Engrailed-2 (EN-2) promoter is associated with increased gene expression and decreased MeCP2 binding in autism cerebellum. Transl. Psychiatry 4, e460. 10.1038/tp.2014.87 25290267 PMC4350522

[B47] Jirtle R. L. Skinner M. K. (2007). Environmental epigenomics and disease susceptibility. Nat. Rev. Genet. 8, 253–262. 10.1038/nrg2045 17363974 PMC5940010

[B48] Jones P. A. (2001). The Role of DNA Methylation in Mammalian Epigenetics. Science 293, 1068–1070. 10.1126/science.1063852 11498573

[B49] Joubert B. R. den Dekker H. T. Felix J. F. Bohlin J. Ligthart S. Beckett E.. (2016). Maternal plasma folate impacts differential DNA methylation in an epigenome-wide meta-analysis of newborns. Nat. Commun. 7, 10577. 10.1038/ncomms10577 26861414 PMC4749955

[B50] Kau A. L. Ahern P. P. Griffin N. W. Goodman A. L. Gordon J. I. (2011). Human nutrition, the gut microbiome and the immune system. Nature 474, 327–336. 10.1038/nature10213 21677749 PMC3298082

[B51] Keil A. Daniels J. L. Forssen U. Hultman C. Cnattingius S. Söderberg K. C.. (2010). Parental autoimmune diseases associated with autism spectrum disorders in offspring. Epidemiology 21, 805–808. 10.1097/EDE.0b013e3181f26e3f 20798635 PMC3115699

[B52] Kiefer J. C. (2007). Epigenetics in development. Dev. Dyn. 236, 1144–1156. 10.1002/dvdy.21094 17304537

[B53] Kratsman N. Getselter D. Elliott E. (2015). Sodium Butyrate attenuates social behavior deficits and modifies the transcription of inhibitory/excitatory genes in the frontal cortex of an autism model. Neuropharmacology 102, 136–145. 10.1016/j.neuropharm.2015.11.003 26577018

[B54] Küpers L. K. Xu X. Jankipersadsing S. A. Vaez A. la Bastide-van Gemert S. Scholtens S.. (2015). DNA methylation mediates the effect of maternal smoking during pregnancy on birthweight of the offspring. Int. J. Epidemiol. 44, 1224–1237. 10.1093/ije/dyv048 25862628 PMC4588868

[B55] Labouesse M. A. Dong E. Grayson D. R. Guidotti A. Meyer U. (2015). Maternal immune activation induces GAD1 and GAD2 promoter remodeling in the offspring prefrontal cortex. Epigenetics 10, 1143–1155. 10.1080/15592294.2015.1114202 26575259 PMC4844233

[B56] Ladd-Acosta C. Hansen K. D. Briem E. Fallin M. D. Kaufmann W. E. Feinberg A. P. (2014). Common DNA methylation alterations in multiple brain regions in autism. Mol. Psychiatry 19, 862–871. 10.1038/mp.2013.114 23999529 PMC4184909

[B57] Lasalle J. M. (2013). Autism genes keep turning up chromatin. OA Autism 1, 14. 24404383 10.13172/2052-7810-1-2-610 PMC3882126

[B58] Lee B. K. Magnusson C. Gardner R. M. Blomström S. Newschaffer C. J. Burstyn I.. (2014). Maternal hospitalization with infection during pregnancy and risk of autism spectrum disorders. Brain. Behav. Immun. 44, 100–105. 10.1016/j.bbi.2014.09.001 25218900 PMC4418173

[B59] Lee K. W. K. Richmond R. Hu P. French L. Shin J. Bourdon C.. (2015). Prenatal exposure to maternal cigarette smoking and DNA methylation: epigenome-wide association in a discovery sample of adolescents and replication in an independent cohort at birth through 17 years of age. Environ. Health Perspect. 123, 193–199. 10.1289/ehp.1408614 25325234 PMC4314251

[B60] Lintas C. Sacco R. Persico A. M. (2016). Differential methylation at the RELN gene promoter in temporal cortex from autistic and typically developing post-puberal subjects. J. Neurodev. Disord. 8, 18. 10.1186/s11689-016-9151-z 27134686 PMC4850686

[B61] Loke Y. J. Hannan A. J. Craig J. M. (2015). The role of epigenetic change in autism spectrum disorders. Front. Neurol. 6:107. 10.3389/fneur.2015.00107 26074864 PMC4443738

[B62] LoParo D. Waldman I. D. (2015). The oxytocin receptor gene (OXTR) is associated with autism spectrum disorder: a meta-analysis. Mol. Psychiatry 20, 640–646. 10.1038/mp.2014.77 25092245

[B63] Machado C. J. Whitaker A. M. Smith S. E. P. Patterson P. H. Bauman M. D. (2015). Maternal immune activation in nonhuman primates alters social attention in juvenile offspring. Biol. Psychiatry 77, 823–832. 10.1016/j.biopsych.2014.07.035 25442006 PMC7010413

[B64] Maezawa I. Jin L. W. (2010). Rett syndrome microglia damage dendrites and synapses by the elevated release of glutamate. J. Neurosci. 30, 5346–5356. 10.1523/JNEUROSCI.5966-09.2010 20392956 PMC5533099

[B65] Malkova N. V. Yu C. Z. Hsiao E. Y. Moore M. J. Patterson P. H. (2012). Maternal immune activation yields offspring displaying mouse versions of the three core symptoms of autism. Brain. Behav. Immun. 26, 607–616. 10.1016/j.bbi.2012.01.011 22310922 PMC3322300

[B66] Mazina V. Gerdts J. Trinh S. Ankenman K. Ward T. Dennis M. Y.. (2015). Epigenetics of autism-related impairment: copy number variation and maternal infection. J. Dev. Behav. Pediatr. 36, 61–67. 10.1097/DBP.0000000000000126 25629966 PMC4318761

[B67] Melemedjian O. K. Tillu D. V. Moy J. K. Asiedu M. N. Mandell E. K. Ghosh S.. (2014). Local translation and retrograde axonal transport of CREB regulates IL-6-induced nociceptive plasticity. Mol. Pain 10, 45. 10.1186/1744-8069-10-45 24993495 PMC4091745

[B68] Nardone S. Sharan Sams D. Reuveni E. Getselter D. Oron O. Karpuj M.. (2014). DNA methylation analysis of the autistic brain reveals multiple dysregulated biological pathways. Transl. Psychiatry 4, e433. 10.1038/tp.2014.70 25180572 PMC4203003

[B69] Nemoda Z. Massart R. Suderman M. Hallett M. Li T. Coote M.. (2015). Maternal depression is associated with DNA methylation changes in cord blood T lymphocytes and adult hippocampi. Transl. Psychiatry 5, e545. 10.1038/tp.2015.32 25849984 PMC4462598

[B70] Ogryzko V. V. Schiltz R. L. Russanova V. Howard B. H. Nakatani Y. (1996). The Transcriptional Coactivators p300 and CBP Are Histone Acetyltransferases. Cell 87, 953–959. 10.1016/S0092-8674(00)82001-2 8945521

[B71] Quilter C. R. Cooper W. N. Cliffe K. M. Skinner B. M. Prentice P. M. Nelson L.. (2014). Impact on offspring methylation patterns of maternal gestational diabetes mellitus and intrauterine growth restraint suggest common genes and pathways linked to subsequent type 2 diabetes risk. FASEB J. 28, 4868–4879. 10.1096/fj.14-255240 25145626

[B72] Richmond R. C. Simpkin A. J. Woodward G. Gaunt T. R. Lyttleton O. McArdle W. L. (2015). Prenatal exposure to maternal smoking and offspring DNA methylation across the life course: findings from the Avon Longitudinal Study of Parents and Children (ALSPAC). Hum. Mol. Genet. 24, 2201–2217. 10.1093/hmg/ddu739 25552657 PMC4380069

[B73] Robinson E. B. St Pourcain B. Anttila V. Kosmicki J. A. Bulik-Sullivan B. Grove J.. (2016). Genetic risk for autism spectrum disorders and neuropsychiatric variation in the general population. Nat. Genet. 48, 552–555. 10.1038/ng.3529 26998691 PMC4986048

[B74] Schmidt R. J. Tancredi D. J. Ozonoff S. Hansen R. L. Hartiala J. Allayee H.. (2012). Maternal periconceptional folic acid intake and risk of autism spectrum disorders and developmental delay in the CHARGE (CHildhood Autism Risks from Genetics and Environment) case-control study. Am. J. Clin. Nutr. 96, 80–89. 10.3945/ajcn.110.004416 22648721 PMC3374734

[B75] Shishido E. Aleksic B. Ozaki N. (2014). Copy-number variation in the pathogenesis of autism spectrum disorder. Psychiatry Clin. Neurosci. 68, 85–95. 10.1111/pcn.12128 24372918

[B76] Shulha P. Houston I. B. Chou H. J. Bharadwaj R. Cheung I. Tushier J. S.. (2012). Human-specific histone methylation signatures at transcription start sites in prefrontal neurons. PLoS. Biol. 10:e1001427. 10.1371/journal.pbio.1001427 23185133 PMC3502543

[B77] Smith S. E. P. Li J. Garbett K. Mirnics K. Patterson P. H. (2007). Maternal immune activation alters fetal brain development through interleukin-6. J. Neurosci. 27, 10695–10702. 10.1523/JNEUROSCI.2178-07.2007 17913903 PMC2387067

[B78] Spiers H. Hannon E. Schalkwyk L. C. Smith R. Wong C. C. Y. O'Donovan M. C.. (2015). Methylomic trajectories across human fetal brain development. Genome Res. 25, 338–352. 10.1101/gr.180273.114 25650246 PMC4352878

[B79] Sultana R. Yu C. E. Yu J. Munson J. Chen D. Hua W.. (2002). Identification of a novel gene on chromosome 7q11.2 interrupted by a translocation breakpoint in a pair of autistic twins. Genomics 80, 129–134. 10.1006/geno.2002.6810 12160723

[B80] Surén P. Roth C. Bresnahan M. Haugen M. Hornig M. Hirtz D.. (2013). Association between maternal use of folic acid supplements and risk of autism spectrum disorders in children. JAMA 309, 570–577. 10.1001/jama.2012.155925 23403681 PMC3908544

[B81] Tang B. Jia H. Kast R. J. Thomas E. A. (2013). Epigenetic changes at gene promoters in response to immune activation *in utero*. Brain. Behav. Immun. 30, 168–175. 10.1016/j.bbi.2013.01.086 23402795

[B82] Tordjman S. Somogyi E. Coulon N. Kermarrec S. Cohen D. Bronsard G.. (2014). Gene × Environment interactions in autism spectrum disorders: role of epigenetic mechanisms. Front. Psychiatry 5:53. 10.3389/fpsyt.2014.00053 25136320 PMC4120683

[B83] Turner T. N. Hormozdiari F. Duyzend M. H. McClymont S. A. Hook P. W. Iossifov I.. (2016). Genome sequencing of autism-affected families reveals disruption of putative noncoding regulatory DNA. Am. J. Hum. Genet. 98, 58–74. 10.1016/j.ajhg.2015.11.023 26749308 PMC4716689

[B84] Vinet É. Pineau C. A. Clarke A. E. Scott S. Fombonne É. Joseph L.. (2015). Increased risk of autism spectrum disorders in children born to women with systemic lupus erythematosus: results from a large population-based cohort. Arthritis Rheumatol. 67, 3201–3208. 10.1002/art.39320 26315754

[B85] Voineagu I. Wang X. Johnston P. Lowe J. K. Tian Y. Horvath S.. (2011). Transcriptomic analysis of autistic brain reveals convergent molecular pathology. Nature 474, 380–384. 10.1038/nature10110 21614001 PMC3607626

[B86] Wang J. Wegener J. E. Huang T. W. Sripathy S. De Jesus-Cortes H. Xu P. (2015). Wild-type microglia do not reverse pathology in mouse models of Rett syndrome. Nature 521, E1–E4. 10.1038/nature14444 25993969 PMC4684952

[B87] Wei L. Vahedi G. Sun H. W. Watford W. T. Takatori H. Ramos H. L.. (2010). Discrete roles of STAT4 and STAT6 transcription factors in tuning epigenetic modifications and transcription during T helper cell differentiation. Immunity 32, 840–851. 10.1016/j.immuni.2010.06.003 20620946 PMC2904651

[B88] Zaretsky M. V. Alexander J. M. Byrd W. Bawdon R. E. (2004). Transfer of inflammatory cytokines across the placenta. Obstet. Gynecol. 103, 546–550. 10.1097/01.AOG.0000114980.404453 14990420

[B89] Zheng F. Wang L. Jia M. Yue W. Ruan Y. Lu T.. (2011). Evidence for association between Disrupted-in-Schizophrenia 1 (DISC1) gene polymorphisms and autism in Chinese Han population: a family-based association study. Behav. Brain Funct. 7, 14. 10.1186/1744-9081-7-14 21569632 PMC3113723

[B90] Zhou J. Ping F. Lv W. Feng J. Shang J. (2014). Interleukin-18 directly protects cortical neurons by activating PI3K/AKT/NF-κB/CREB pathways. Cytokine 69, 29–38. 10.1016/j.cyto.2014.05.003 25022959

[B91] Zhu L. Wang X. Li X. L. Towers A. Cao X. Wang P.. (2014). Epigenetic dysregulation of SHANK3 in brain tissues from individuals with autism spectrum disorders. Hum. Mol. Genet. 23, 1563–1578. 10.1093/hmg/ddt547 24186872 PMC3929093

[B92] Zijlstra G. J. Ten Hacken N. H. T. Hoffmann R. F. van Oosterhout A. J. M. Heijink I. H. (2012). Interleukin-17A induces glucocorticoid insensitivity in human bronchial epithelial cells. Eur. Respir. J. 39, 439–445. 10.1183/09031936.00017911 21828034

